# Consequences of peritonism in an Emergency Department setting

**DOI:** 10.1186/1757-7241-20-S2-P11

**Published:** 2012-04-16

**Authors:** Thomas Bjørsum-Meyer, Thomas Andersen Schmidt

**Affiliations:** 1 Emergency Department, Holbaek Hospital, Denmark

## Background

In patients referred to the Emergency Department (ED) with abdominal pain, it is crucial to determine the presence of peritonism in order to allow for appropriate handling and subsequent referral to other wards.

Peritonism is indicative of the need for acute surgical intervention.

Thus, patients with either 1) referred tenderness, 2) tenderness from percussion, 3) rebound tenderness or 4) combinations hereof need admission to the Department of Surgery, while patients without peritonism in general terms do not.

We aimed to assess the incidence of peritonism and the handling of patients admitted to the ED with abdominal pain. To our knowledge this is the first such evaluation in a Danish setting.

## Methods

We evaluated 1270 patients consecutively admitted to the ED and 128 (10%) patients presented with abdominal pain. Fifty-one patients were found to have signs of peritonism, 77 patients did not. Data were collected from Triage and Nursing protocols and subsequently also patient charts.

## Results

Among 128 patients presenting with abdominal pain in the ED, 40% (N=51) were found to have signs of peritonism and as suspected nearly all 90% (N=46) were admitted to the Department of Surgery. 24% (N=11) of patients with signs of peritonism and admission to the Department of Surgery underwent acute surgery in terms of laparotomy/laparoscopy, 13% (N=5) of patients without signs of peritonism but admission to the Department of Surgery were subjected to acute surgery (p = 0.27).

Patients with signs of peritonism were younger 34 vs. 52 years (p = 0.05) and were hospitalized at the Department of Surgery for a shorter time 38.2 vs. 95.3 hours (p=0.05).

No differences were seen with regard to duration of stay in the ED or surgical supervision in the ED between groups with and without signs of peritonism.

## Conclusion

Patients with abdominal pain represent a significant share in the ED.

Our study highlights the need for improving skills in abdominal examination and recognition of true peritonism for correct visitation and preparation for acute surgery. In order to improve skills we recommend more supervision from doctors with substantial surgical experience and video-assisted education.

